# Moderate burden amongst caregivers posthip arthroscopy linked to younger caregiver age and task load: A cross‐sectional survey study

**DOI:** 10.1002/ksa.12414

**Published:** 2024-08-15

**Authors:** Yoan Bourgeault‐Gagnon, Mansi Patel, Madison Walker, Hassaan Abdel Khalik, Andrew Duong, Nicole Simunovic, Olufemi R. Ayeni, The Caregiver Burden Survey Investigators

**Affiliations:** ^1^ Division of Orthopaedic Surgery, Department of Surgery McMaster University Hamilton Ontario Canada

**Keywords:** caregiver, caregiver burden, hip arthroscopy, outpatient surgery, rehabilitation

## Abstract

**Purpose:**

To evaluate the burden experienced by primary informal caregivers of patients who have undergone hip arthroscopy and to identify factors that predict increased caregiver burden.

**Methods:**

A cross‐sectional study was conducted at a single academic hospital centre, enroling caregivers of patients who underwent hip arthroscopy between November 2018 and November 2023. Caregiver burden was assessed using the Caregiver Burden Inventory (CBI) survey. Multivariable linear regression models were used to identify predictors of caregiver burden, with the global CBI score serving as the primary outcome measure. Secondarily, open‐ended survey questions were analyzed qualitatively to elucidate specific challenges and facilitators of caregiving, as reported by the caregivers themselves.

**Results:**

The study involved 99 eligible caregivers (mean [standard deviation] age; 47 [11] years), 58% were female, and 85% were relatives of the patient. The median global CBI score was 13.0 (interquartile range: 8.0–22.4), indicating a moderate burden. Regression analyses demonstrated that younger caregiver age and a higher number of caregiving tasks were significant predictors of increased global burden. Additionally, nonweightbearing status of patients, female gender of caregivers and working full‐time statistically significantly increased specific dimensions of caregiver burden.

**Conclusion:**

This study highlights the meaningful burden faced by caregivers of patients undergoing hip arthroscopy, despite its minimally invasive nature and outpatient setting. Identified risk factors such as younger caregiver age, female gender of the caregiver, nonweight‐bearing status and increased caregiving tasks suggest targeted areas for intervention. The qualitative analysis revealed that caregivers struggle with time management and physical and emotional strain, yet better communication and practical support from healthcare teams could help to alleviate these challenges.

**Level of Evidence:**

Level IV, prognostic study.

**Abbreviations**
CBI: Caregiver Burden Inventory
CI: confidence interval
IQR: interquartile range
SD: standard deviation

## INTRODUCTION

Hip arthroscopy is increasingly used to treat acetabular labral tears, chondral injuries and femoroacetabular impingement syndrome. In the United States, hip arthroscopy volumes increased fivefold from 2004 to 2016, driven by expanded indications, favourable evidence and high‐volume surgeons [31]. Similar trends are seen in Spain, with incidence rates rising from 0.14 per 100,000 in 1998 to 4.09 in 2018, and projections suggest a further 150% increase by 2030 [22].

As a minimally invasive procedure, hip arthroscopy typically allows shorter recovery times and can be performed on an outpatient basis [4]. Nonetheless, hip arthroscopy differs from other ambulatory arthroscopic procedures in several ways, including rehabilitation protocols that may involve prolonged periods of restricted weightbearing, its utilization in both adolescent and older patient populations, as well as being performed on relatively more complex patients from a psychosocial perspective [20, 27, 28, 32]. As a result, recovery from hip arthroscopy requires significant aid from informal caregivers such as family and friends to support through the recovery period.

Interest in caregiver burden in orthopaedic surgery has increased, particularly with more procedures being performed on an outpatient basis [17]. Caregivers face emotional, physical and financial stressors often overlooked in the literature [19]. Previous studies have mostly focused on caregivers of hip fracture or arthroplasty patients, who experience higher morbidity, but there is little research on caregivers of hip arthroscopy patients [1, 16, 17]. This may stem from the fact that these patients are younger and have fewer comorbidities compared to other high‐volume orthopaedic populations [3]. Consequently, research on hip arthroscopic surgery has typically focused on patient‐centred outcomes and ignored the impact on the patient's family and informal caregivers, which may leave them unprepared for the recovery process and the associated burden of postoperative care.

This cross‐sectional survey study aimed to evaluate the burden on informal primary caregivers of hip arthroscopy patients and to identify factors predicting increased burden. Secondarily, it also sought to identify positive and negative contributors to burden. It was hypothesized that caregivers face a moderate burden when providing postoperative care.

## MATERIALS AND METHODS

### Study design

This is a survey study reported using the Strengthening the Reporting of Observational Studies in Epidemiology checklist for cross‐sectional studies [12]. All procedures were approved by the Hamilton Integrated Research Ethics Board (project # 4970).

### Outcome measures and questionnaire development

The questionnaire was developed by a team of orthopaedic surgeons, expert researchers in the field of hip arthroscopy, as well as past literature [1, 7, 15, 23, 35]. The survey captured data on caregiver and care‐recipient demographics, the challenges and facilitators of caregiving (File S1) and the CBI (File S2). The CBI is a valid and reliable questionnaire measuring caregiver burden that consists of five components, including (1) time dependence burden: the time demands and constraints placed on caregivers; (2) developmental burden: captures the caregivers' feelings of being developmentally ‘off‐time’ compared to peers; (3) physical burden: pertains to the strain on caregivers' health, strength and energy; (4) social relationship burden: involves the role conflict experienced by caregivers and (5) emotional burden: includes negative feelings towards care receivers [6, 8, 21]. Each component includes 4–5 questions which are all graded from 0 to 4 (0—*Never*; 1—*Rarel*y; 2—*Sometimes*; 3—*Quite Frequently*; 4— *Nearly Always*).

Scores for each component were calculated by summing the grades for each question. A global CBI score (or ‘total adjusted burden score’) was calculated by summing the total scores for each component [21]. For the global CBI score, the physical burden component was multiplied with a factor of 1.25 to have the same weight as the other components [21]. A higher global score reflects a greater burden on the primary caregiver, ranging from 0 (no burden) to 100 (highest burden). Previously published thresholds identify a score between 24 and 35 as indicative of a substantial burden, suggesting a need for respite care, while a score of 36 or more signals a risk of ‘burning out’ [10, 30]. Scores between 12 and 23 were defined to represent ‘moderate’ burden and scores of 11 and below represented little to no burden.

To assess the secondary outcomes, participants were asked to list three challenges to caregiving and three ways that healthcare teams can better support caregivers, in two open‐ended questions.

### Settings and participants

Participants were consecutively recruited from an orthopaedic clinic at McMaster Children's Hospital and McMaster University Medical Center. Participants eligible to participate in the study were aged 18 or over, self‐identified primary caregivers of any patient who had undergone hip arthroscopic surgery (i.e., provided the majority of care to the patient), and able to read, write, and understand English. All surgeries were performed by a single surgeon as ambulatory surgeries. A standardized rehabilitation protocol was given to every included patient, including weight‐bearing instructions. Caregivers providing informal care to multiple individuals or receiving any form of financial compensation for the patient's care were ineligible to participate.

### Questionnaire administration

The data was collected via a paper version of the survey from November 2018 to November 2023. Research assistants initially screened participants before their postoperative follow‐up appointment (i.e., any follow‐up appointment up to 6 weeks postoperatively). During the follow‐up appointment, a research assistant met with the primary caregiver to obtain written informed consent for participation in the study. Then, the questionnaire was administered on paper to the caregiver, and the data was subsequently transferred and recorded in a spreadsheet by the research assistant. Each participant was afforded the opportunity to complete their questionnaire in private (i.e., away from the patient they were caring for). To maximize the response rate, surgeons initially introduced the study to patients and their caregivers and the questionnaire was completed concurrently with a routine postoperative follow‐up.

### Sample size

The primary outcome was the CBI component of the survey. Using an *α* value of 0.05, power of 90% and an effect size of 0.38, reported by past literature, the required sample size was 77 [18]. However, the target sample size was increased to 100 to account for possible missing data and to ensure the planned regression analyses were not overfitted and were reliable. The sample size was calculated using G*Power (Version 3.1.9.6; 2007).

### Statistical analysis

Continuous outcomes and variables were reported as means (standard deviation [SD]), if normally distributed, or medians (interquartile range [IQR]), if not. Categorial data was reported as proportions. A Shapiro–Wilk test was used to test for normal distribution of continuous variables.

Six multivariable linear regressions were performed to evaluate demographic and caregiving characteristics associated with caregiver burden, measured by the five individual components and global CBI scores (higher score = greater burden). Ten independent variables were selected a priori, hypothesized to be associated with caregiver burden. To ensure that the regression models were not overfitted and were reliable, 10 participants were required for each independent variable/factor in the regression [2]. Guided by past literature and clinical experiences, the direction of effect for each variable was hypothesized [1, 7, 15, 25, 26] (Supporting Information S1: Table S1). A multiple imputation by chained equations was performed to impute values for the missing data. The primary regression analyses were based on 10 multiple imputed datasets, and results were combined using Rubin's combination rules [29, 34]. The estimates from imputed and complete case analysis were analysed to ensure consistency of results. Measures of association as *β* values and associated 95% confidence intervals (CIs) were reported. An independent factor was considered statistically significant if it had a *p* < 0.05 in the final multivariable regression model. All analyses were conducted using RStudio (v4.1.0; R Core Team 2021).

Responses to open‐ended questions were evaluated using content analysis to identify patterns. A constant comparative approach was used to develop a coding template including categories of facilitators and barriers to caregiving and proportion of respondents endorsing each [14]. Findings were narratively summarized. Moreover, representative quotes for each category were captured.

## RESULTS

### Caregiver and care receiver characteristics

A total of 101 caregivers were enroled due to the availability of an additional participant available on the final day of recruitment. Of these, 99 caregivers were found to be eligible and were included in the analysis, whereas two participants were later found to be ineligible as they were providing care to other individuals as well. The mean age of included caregivers was 47 (SD 11) years with 58% (57/98) being female. Most caregivers were relatives of the patient (85%, 81/95) and were White/Caucasian (86%, 85/99). The most common highest attained level of education for caregivers was earning a college diploma or trade/vocational training (44%, 42/96), followed by earning a bachelor's degree (23%, 22/96). Employment status of caregivers was primarily comprised of full‐time (66%, 64/97) or part‐time (14%, 14/97) employment. Slightly more than half of caregivers were parents of children under 18 years of age (54%, 52/97). Sole caregivers comprised slightly less than half of all caregivers (42%, 41/98). Most caregivers lived with the patient (88%, 86/98) (Table 1).

**Table 1:** Demographic characteristics of caregivers.

Characteristic	*N*	*n* (%)
Age (mean, SD)	98	47 (11)
Gender	98	
Female		57 (58)
Male		41 (42)
Relationship to care recipient	95	
Relative		81 (85)
Nonrelative		14 (15)
Marital status	99	
Married		79 (80)
Divorced or partner		13 (13)
Single		7 (7)
Race	99	
White/Caucasian		85 (86)
South Asian		4 (4)
Black/African/Caribbean		4 (4)
Native/Aboriginal		2 (2)
Hispanic/Latino		2 (2)
South East Asian		2 (2)
Highest level of education	96	
Less than high school		2 (2)
High school/equivalent		14 (15)
College diploma or trade/vocational training		42 (44)
Bachelor's degree		22 (23)
Masters/professional degree		14 (15)
Doctorate		2 (2)
Employment status at time of survey	97	
Employed—full time		64 (66)
Employed—part time		14 (14)
Retired		10 (10)
Unable to work		5 (5)
Unemployed		3 (3)
Student		1 (1)
Employment status before being a caregiver	99	
Employed—full time		68 (69)
Employed—part time		13 (13)
Retired		9 (9)
Unable to work		5 (5)
Unemployed		3 (3)
Student		1 (1)
Caregiver's parental status	97	
Yes, any children under 18 years old		52 (54)
No children under 18 years old		45 (46)
Sole caregiver to the patient	98	
No—other unpaid caregiver (e.g., relative)		55 (56)
Yes		41 (42)
No—other paid caregiver (e.g., PSW, nurse)		2 (2)
Previous care provided for recipient for other medical conditions	95	59 (62)
Caregiver lives with recipient	98	86 (88)
Distance between caregiver's and recipient's habitation (km)^a^	12	
0–15		5 (42)
15–30		3 (25)
30–50		1 (8)
50+		3 (25)

Abbreviations: km, kilometres; PSW, personal support worker; SD, standard deviation.

^a^ Only answered by caregivers that did not already live with the patient.

The median age of patients was 30 (IQR: 18–40) years with 75% (74/99) of patients being female. Preoperative activity levels were mostly high (40%, 39/97) or medium (39%, 38/97), quantified as more than 3 h of weekly exercise or between 1 and 3 h of weekly exercise, respectively. The most common comorbidity was back pain (27%, 27/99) though half of patients did not present with any additional comorbidities (52%, 51/99) (Table 2).

**Table 2:** Demographic and clinical characteristics of care receivers (patients).

Characteristic	*N*	*n* (%)
Age (median, IQR)	96	30 (18, 40)
Gender	99	
Female		74 (75)
Male		25 (25)
Comorbidities	99	
Back pain		27 (27)
Previous lower extremity injury		13 (13)
Depression		11 (11)
Osteoarthritis		7 (7)
Anaemia		5 (5)
Other^a^		26 (26)
Number of comorbidities	99	
0		51 (52)
1		18 (18)
2		14 (14)
3		5 (5)
4		4 (4)
5+		7 (7)
Procedure performed^b^	98	
Simple arthroscopy		86 (88)
Labral repair		66 (67)
Osteochondroplasty		23 (23)
Loose body removal		1 (1)
Other^c^		4 (4)
Unknown		1 (1)
Complications	99	
Nausea		2 (2)
Fall (uncomplicated)		1 (1)
Scarring		1 (1)
Severe persistent pain		1 (1)
Inner ear infection		1 (1)
Urinary retention		1 (1)
Opioid side effects (undefined)		1 (1)
Hospitalization		1 (1)
Rash		1 (1)
Allergic reaction		1 (1)
Vasovagal syncope		1 (1)
Patient's fitness level before surgery (h/week)	97	
High (>3)		39 (40)
Medium (1–3)		38 (39)
Low (<1)		20 (21)
Time since procedure (days) (median, IQR)	92	11.0 (10.0, 12.0)
Weight‐bearing status at the time of survey	98	
Nonweight bearing		74 (76)
Partial weight bearing		24 (24)
Full weight bearing		0 (0)
Mobility aids	99	
Two crutches		91 (91)
Wheelchair		19 (19)
Walker		13 (13)
One crutch		3 (3)
Cane		3 (3)
Other^d^		3 (3)
None (ambulatory)		1 (1)

Abbreviation: IQR, interquartile range.

^a^ Postconcussion syndrome, anxiety, Raynaud's syndrome, Ehler–Danlos syndrome, postural orthostatic tachycardia syndrome, posttraumatic stress disorder, fibromyalgia, sensorineural hearing loss, irritable bowel disease, celiac disease, spondylitis, Graves' disease, psoriatic arthritis, anorexia nervosa, bipolar disorder, borderline personality.

^b^ As reported by the patient.

^c^ Labral reconstruction, psoas tendon release and ‘not reported’.

^d^ Knee walker, bath bench, shower chair, raised toilet seat, stair chair and bathtub handle.

The caregiver survey was conducted at a median time of 11 (IQR: 10–12) days postoperatively. Seventy‐six percent (74/98) of patients were nonweightbearing at the time of the survey, with 91% (91/99) requiring two crutches (Table 2).

Most caregivers provided care for patients for a period ranging between 1 week and 1 month (87%, 85/98) at the time of the survey (Table 3). Furthermore, most caregivers needed to provide care more than once a day during this period of time (88%, 84/96). The three most common tasks caregivers provided to the patients were transportation (96%, 95/99), health care (96%, 95/99) and household chores (94%, 93/99). Almost half of caregivers noted that the care recipient required assistance with three or more personal tasks (49%, 47/96).

**Table 3:** Caregiving components.

Characteristic	*N*	*n* (%)
Duration of care provided to recipient	98	
<1 week		7 (7)
1 week to 1 month		85 (87)
1 month to 6 months		3 (3)
More than 2 years		3 (3)
Frequency of provided care to recipient	96	
More than once a day		84 (88)
Once every day		4 (4)
A few times per week		3 (3)
A few times per month		2 (2)
Once a month or less		3 (3)
Caregiver moved to be closer to recipient^a^	70	2 (3)
Caregiving tasks provided to recipient	99	
Transportation		95 (96)
Health care		95 (96)
House making chores		93 (94)
Personal care tasks		82 (83)
Emotional support		76 (77)
Supervision		46 (46)
Caring for children or other dependants		37 (37)
Other^b^		7 (7)
Caregiving critical factors	96	
Care recipient requires assistance with three or more personal care tasks		47 (49)
Caregiving will likely continue indefinitely		11 (11)
I was recently hospitalized (or had a recent health crisis)		5 (5)
My income is at or below federal poverty level		3 (3)
Care recipient is at risk for institutionalization		2 (2)
None of the statements apply to me		35 (36)
Prefer not to answer		6 (17)

a If not already living with the patient.

^b^ ‘Fetching things’, ‘benefits coverage’, ‘medication and equipment’, ‘purchase/rentals’, ‘physiotherapy’, ‘mental health support’, ‘everything’ and ‘taking care of farm animals’.

### Primary outcome

The median global CBI score amongst the caregiver population was 13.0 (IQR: 8.0–22.4) out of 100, which was indicative of moderate burden. Notably, 13 (13%) of caregivers had a score between 24 and 36, indicating a potential need for respite, while 7 (7%) had scores exceeding 36, placing them at risk of ‘burning out’. When evaluating individual CBI components, the most increased CBI dimension (median, IQR) was the time dependency score (9.0, IQR: 5.0–12.0 out of 20) followed by the physical health (2.0, IQR: 0.0–5.0 out of 16), development (1.0, IQR: 0.0–4.0 out of 20), social relationships (0.0, IQR: 0.0–1.0 out of 20) and emotional health (0.0, IQR: 0.0–0.0 out of 20) scores.

In the multivariable regression model assessing the global CBI as the dependant variable and the characteristics of the caregiver, care receiver and the caregiving episode as independent variables, the predicting factors accounted for 20.6% of the variability in the global CBI (adjusted *R* ^2^ = 0.206). Decreasing age of the caregiver was statistically significantly associated with a higher global burden score (*β* = −0.277; 95% CI = −0.513 to −0.042; *p* = 0.021). Additionally, the greater the number of caregiving tasks provided was statistically significantly associated with a higher burden (*β* = 2.20; 95% CI = 0.537–3.86; *p* = 0.008). Other predictors were not statistically significant (Table 4).

**Table 4:** Association between the global Caregiver Burden Inventory score and characteristics of the caregiver, the care receiver and the caregiving episode.

Predictor	*β*	95% CI	*p* Value
Caregiver age	−0.277	−0.513, −0.042	**0.021**
Caregiver gender			
Male	–	–	
Female	3.62	−1.17, 8.41	0.14
Frequency of care provided			
More than once a day	–	–	
Once a day or less	−5.75	−13.2, 1.67	0.13
Caregiver has one or more children < 18 years of age			
Yes	–	–	
No	−2.17	−7.02, 2.67	0.37
Caregiver's employment status			
Currently working full‐time or studying	–	–	
Not currently working full‐time	3.96	−1.43, 9.35	0.15
Patient's fitness level before surgery			
Low (<1 h/week) to medium (1–3 h/week)	–	–	
High (>3 h/week)	−0.382	−5.29, 4.53	0.88
Weight‐bearing status			
Nonweight bearing	–	–	
Partial weight bearing	−4.97	−10.4, 0.432	0.071
The caregiver lives with the recipient			
Yes	–	–	
No	−0.164	−8.25, 7.92	0.97
Number of caregiving tasks provided	2.26	0.610, 3.91	**0.008**
Number of comorbidities	1.06	−0.222, 2.34	0.10
Adjusted *R* ^*2*^ = 0.206			

*Note*: Bolded *p* values = *p* < .05.

Abbreviation: CI, confidence interval.

When assessing more specific categories of the CBI, the set of predictor variables was shown to have a ‘weak’ association with the CBI time dependency, development, social relationships, physical or emotional health scores (adjusted *R* ^2^ = 0.218, 0.133, 0.126, 0.06, −0.025, respectively).

Increasing caregiver age (*β *= −0.095; 95% CI = −0.183 to −0.007; *p* = 0.035) and partial or full weightbearing status were statistically significantly associated with less time dependency burden (*β* = −2.08; 95% CI = −4.11 to −0.057; *p* = 0.044). Conversely, more caregiver tasks were associated with significantly higher (i.e., worse) time dependency scores (*β* = 1.12; 95% CI = 0.503–1.73; *p* < 0.001) (Supporting Information S1: Table S2). Younger caregiver age (*β* = −0.090; 95% CI = −0.169 to −0.011; *p* = 0.027) and being female (*β *= 1.80; 95% CI = 0.194–3.40; *p* = 0.028) were associated with increased caregiver burden from a development perspective (Supporting Information S1: Table S3). Caregivers not currently working full‐time exhibited a significantly higher (worse) CBI social relationships score compared to those working full‐time or studying (*β *= 1.34; 95% CI = 0.309–2.37; *p* = 0.012) (Supporting Information S1: Table S4). No other independent variables demonstrated significant relationships with the CBI physical or emotional health Scores (Supporting Information S1: Tables S5 and S6).

### Secondary outcomes

Qualitatively, the three most common factors reported as rendering caregiving challenging included time management (52.5%, 52/99), having to take care of other family members in addition to the patient (20.2%, 20/99), and the physical and emotional strain incurred through the act of caregiving (18.2%, 18/99) (Table 5, Supporting Information S1: Table S7).

**Table 5:** A summary of the most reported factors that make caregiving challenging.

Reported factors	*n* (%)^a^
Time management challenges	52 (52.5)
Taking care of other family members	20 (20.2)
Physical and emotional strain of caregiving	18 (18.2)
Additional responsibilities and task overload	15 (15.2)
Providing emotional support, coaching and patient's mood management	15 (15.2)
Home environment challenges	13 (13.1)
Personal health and limitations of caregivers	10 (10.1)
Managing medications and medical equipment	9 (9.1)
Lack of information or education on management	7 (7.1)
Challenges with personal hygiene and intimacy	7 (7.1)
Sleep disruption	6 (6.1)
Financial and logistical challenges	4 (4.0)
Challenges with hospital discharge and travel home	3 (3.0)
Isolation and lack of support	2 (2.0)

a Eighty‐one caregivers (81.8%, 81/99) provided at least one answer.

One‐fifth of caregivers reported that more detailed communication and information transfer could improve the caregiving experience (19.2%, 19/99). Approximately 10.1% (10/99) of caregivers felt that extended hospital admission may reduce caregiver burden. Similarly, 9.1% (9/99) of caregivers felt that access to homecare assistance and services may improve the caregiver experience (Table 6, Supporting Information S1: Table S7).

**Table 6:** Reported ways that the healthcare team can facilitate better caregiving.

Reported factors	*n* (%)^a^
More detailed communication and information transfer in general	19 (19.2)
Extended hospital stays	10 (10.1)
Facilitated access to home care assistance and services	9 (9.1)
More education on physical rehabilitation, restrictions and medical equipment	8 (8.1)
Facilitated access to medical equipment	8 (8.1)
More education on medication	7 (7.1)
Financial and economic assistance	5 (5.1)
Optimize discharge	4 (4.0)
Better efficiency of care and follow‐up	3 (3.0)
Encouragement for patient cooperation	1 (1.0)
Other/unrelated^b^	10 (10.1)

a Forty‐seven caregivers (47.5%, 47/99) gave at least one answer.

^b^ Refer to Supporting Information S1: Table S7.

## DISCUSSION

The key findings of this research indicated that caregivers of hip arthroscopy patients experienced moderate burden, with 7% at risk of burnout. Factors associated with an overall increase in caregiver burden included younger patient age, as well as an increased number of caregiving tasks. These findings partially confirmed the initial hypotheses, linking increased burden with younger age, female gender and an increasing number of caregiving tasks, but unexpectedly, full‐time employment was associated with a lower burden, while the other five evaluated factors showed no significant association. The main challenges identified were related to time management, caring for multiple family members, and physical and emotional strain.

Although hip arthroscopies are mostly ambulatory, caregivers still experience strain, although seemingly less than those caring for patients with other musculoskeletal conditions like hip fractures. For example, Ariza‐Vega et al. reported that about 50% of caregivers perceived a high level of burden (≥7 points on the Caregiver Strain Index) in the first month posthip fracture surgery, which is substantially higher than the 20% observed in the current study [1]. Similarly, Lin et al. reported a mean Lee and Wu's caregiver burden rating scale score of 29.89 (SD 11.72) out of 57, demonstrating substantial burden 1 week after hospital discharge following hip fracture surgery [15]. In a population of primary caregivers of patients with spinal cord injuries, Maitan et al. reported a mean total CBI‐spinal cord injury of 43.40 (SD 17.55), which is also substantially worse than the one observed in the studied caregiver population [18]. Studying caregiver burden in similar ambulatory procedures, like meniscal repair, could provide further insights.

The impact of younger caregiver age on burden aligns with previous studies, including a meta‐analysis by Pinquart and Sörensen, showing younger caregivers experience more stress [25]. Similarly, a prospective study by Liu et al. found younger caregiver age linked to poor mental health in hip fracture caregivers [16]. Moreover, results showing higher burden in female caregivers are consistent with those from Pinquart and Sörensen [25]. and several other studies [33]. The findings concerning the number of caregiving tasks also align with those reported by Pinquart and Sörensen [26]. Lastly, although no studies directly assessing the caregiver's working status like in this study were found, the authors hypothesize that working may have beneficial effects due to an improved sense of accomplishment, increased access to social interactions, and respite from caregiving.

These findings can assist orthopaedic departments identify at‐risk caregivers, particularly with the current emphasis on ambulatory care and services [9, 13, 24]. Understanding these risks can inform strategies to ease the postoperative course and enhance satisfaction. Of note, a previous clinical trial on hip fracture outcomes highlighted the significant influence of caregiver mental health on patient recovery [16].

Importantly, caregivers highlighted the need for better communication about their roles and perioperative guidelines, which was consistent with previous hip fracture and ambulatory surgery research [1, 23]. Including primary caregivers in preoperative appointments and providing an overview of their role during counselling could help address these issues. Additionally, using technology and online preprogrammed conversational agents (chatbots) to improve communication and patient education is gaining interest in orthopaedics [5]. Interestingly, a chatbot developed to help for postoperative counselling of hip arthroscopy patients showed an 80% satisfaction rate [11]. The authors believe that a similar approach aimed at assisting both patients and caregivers in the preoperative and postoperative process could be beneficial. Future studies assessing the impact of implementing such strategies on caregiver burden and satisfaction, as well as studies clarifying the relationship between caregiver burden and patient outcomes would be valuable. Ultimately, improving patient communication tools along with enhancing access to medical and rehabilitation equipment could be low‐hanging fruits that improve the ambulatory care process in orthopaedic and hip preservation surgery.

### Limitations

This study assessed only the early postoperative caregiver burden, with evaluations occurring at a median of 11 days after surgery. This lack of hindsight and perspective amongst the caregivers responding to the survey may have influenced their answers, potentially overlooking some long‐term impacts. For example, the relatively low reporting of financial concerns by caregivers may be a result of this short‐term assessment and could be underestimated. Therefore, these results cannot establish long‐term conclusions, although we expect caregiver burden to be most prominent in the early postoperative period. Second, while the CBI has been validated for multiple conditions, including musculoskeletal pathologies [8], it has not been specifically validated in a hip arthroscopy context. As a result, the score may underestimate the burden related to certain dimensions, which were initially conceived for caregivers of patients suffering from Alzheimer's disease. From a cultural standpoint, the majority of patients identified as Caucasian, which limits the applicability of findings to patients of different backgrounds. Similarly, the study was completed at one institution in Ontario, Canada, and the results may not be generalizable to other regions or countries. Finally, the number of participants who declined to participate or their reasons for declining were not tracked, which could theoretically have introduced a selection bias in the sample assessed.

## CONCLUSION

This study highlights the meaningful burden faced by caregivers of patients undergoing hip arthroscopy, despite its minimally invasive nature and outpatient setting. Identified risk factors include younger caregiver age, female gender of the caregiver, nonweight‐bearing status of the patient and increased caregiving tasks. The qualitative analysis revealed that caregivers struggle with time management and physical and emotional strain, yet better communication and practical support from healthcare teams could alleviate these challenges. These results underscore the need for tailored strategies to improve caregiver and patient outcomes.

## AUTHOR CONTRIBUTIONS

The Methods Centre at McMaster University (Olufemi R. Ayeni, Nicole Simunovic, Andrew Duong, Mansi Patel) coordinated the study. The Methods Centre was responsible for programming and maintaining the study database, data validation and data analyses. Yoan Bourgeault‐Gagnon, Mansi Patel, Madison Walker and Hassaan Abdel Khalik wrote the first draft of the manuscript, and the Writing Committee made revisions. Mansi Patel and Yoan Bourgeault‐Gagnon performed all analyses. The Research Assistants were responsible for recruitment and survey administration to participants.

## The Caregiver Burden Survey Investigators

*Writing Committee* (*McMaster University*): Yoan Bourgeault‐Gagnon MD, FRCSC; Mansi Patel MSc; Madison Walker MD; Hassaan Abdel Khalik MD, MMI; Andrew Duong MSc; Nicole Simunovic MSc; Olufemi R. Ayeni MD, PhD, FRCSC. *Methods Centre* (*McMaster University*): Olufemi R. Ayeni MD, PhD, FRCSC (Principal Investigator), Mansi Patel MSc (Statistician), Nicole Simunovic MSc (Research Manager), Andrew Duong MSc (Project Manager). *Co‐Investigators* (*McMaster University*): Jeffrey Kay MD, MSc, FRCSC; Madison Walker MD; Nancy Zhang MD. *Research Assistants* (*McMaster University*): Naziha N. Ali BSc (Cand); Hanu Chaudhari MD; Larissa Maini MD; Vireshwar A. Jagdeo BSc (Cand); Victor Mitea BSc (Cand); Milin Patel BHSc; Nathasha Rajapaksege BSc (Cand); Anish Samanthapudi BSc (Cand); Lauren Sparling BHSc; Prushoth Vivekanantha BMSc; Michael Wu BSc.

## CONFLICTS OF INTEREST STATEMENT

Olufemi R. Ayeni is on the speaker bureau for Stryker Canada, Tier 2 Canada Research Chair in Joint Preservation and President/Owner of Notch Academy. The remaining authors declare no conflict of interest.

## ETHICS STATEMENT

Ethical approval for this study was granted by the Hamilton Integrated Research Ethics Board (HIREB #4970). Written informed consent was obtained from all participants.

## Supporting information

Supporting information.

Supporting information.

Supporting information.

## Data Availability

The data that support the findings of this study are not openly available and are available from the corresponding author upon reasonable request.
