# Supplementary material for: Moderate burden amongst caregivers posthip arthroscopy linked to younger caregiver age and task load: A cross‐sectional survey study
Source: Knee Surg Sports Traumatol Arthrosc. 2024 Aug 15;33(2):728–38. doi: 10.1002/ksa.12414 (PMC11792098; doi:10.1002/ksa.12414)
Supplement: Supplementary file 1 — Supporting information. [file KSA-33-728-s003.pdf]

Authors: The Caregiver Burden Survey Investigators
Corresponding Author: Dr. Olufemi R. Ayeni, McMaster University, ayenif@mcmaster.ca

Online Resource 1

Hip Arthroscopy – Caregiver Demographics Survey

1. Caregiver's:	a. Age	_______________ years
	b. Gender	☐ Male<br>☐ Female<br>☐ Other: _____________<br>__
2. Care recipient's:	a. Age	_______________ years
	b. Gender	☐ Male<br>☐ Female<br>☐ Other: ______________<br>_____
3. Caregiver's relationship to care recipient	☐ Relative<br>☐ Non-Relative	
4. Caregiver demographics:	a. Marital status	☐ Married<br>☐ Single<br>☐ Other _______________
	b. Race	☐ South Asian<br>☐ East Asian<br>☐ White/Caucasian<br>☐ Black/African/Caribbean<br>☐ Native/Aboriginal<br>☐ Hispanic/Latino<br>☐ Other: _______________
	c. Highest level of education. If currently enrolled, select the highest degree received	☐ Less than High School<br>☐ High School/Equivalent (ie. GED)<br>☐ College Diploma or Trade/ Vocational Training<br>☐ Bachelor's Degree<br>☐ Masters/Professional Degree<br>☐ Doctorate

5. Are you the only person providing care to the recipient?	☐ No – other unpaid caregiver (eg: relative)<br>☐ No – other paid caregiver (eg: PSW, nurse)<br>☐ Yes	
6. Duration of Care	a. How long have you provided care to the patient, related to the condition treated with arthroscopy?	☐ < 1 week<br>☐ 1 week – 1 month<br>☐ 1 month – 6 months<br>☐ 6 months – 1 year<br>☐ 1 year – 2 years<br>☐ More than 2 years
	b. Did you provide care to the patient before hip arthroscopy surgery?	☐ Yes<br>☐ No
	c. Have you provided care for this patient for any other medical conditions?	☐ Yes<br>☐ No
7. How often do you provide care to the recipient?	☐ More than once a day<br>☐ Once every day<br>☐ A few times per week<br>☐ A few times per month<br>☐ Once a month or less	
8. Habitation	a. Do you live with the care recipient? [if yes, skip to 8c]	☐ Yes<br>☐ No
	b. How far do you currently live from the recipient?	☐ 0-15 km<br>☐ 15-30 km<br>☐ 30-50 km<br>☐ 50+ km
	c. Did you move to be closer to the patient?	☐ Yes<br>☐ No
9. Is there anyone you can call in an emergency to fill in for you as caregiver?	☐ Yes<br>☐ No	
10. Do you have any children under the age of 18?	☐ Yes<br>☐ No	
11. Are you currently employed?	☐ Employed – full time<br>☐ Employed – part time<br>☐ Retired<br>☐ Unable to work (ie. Disability)<br>☐ Unemployed<br>☐ Student	

12. What was your employment status before you began providing care to the patient?	☐ Employed – full time<br>☐ Employed – part time<br>☐ Retired<br>☐ Unable to work (ie. Disability)<br>☐ Unemployed<br>☐ Student
13. Which tasks do you assist the care recipient with (select all that apply)?	☐ Personal care tasks (bathing, dressing, moving from one place to another, dressing, going to the washroom, etc)<br><br>☐ Housemaker chores (shopping, preparing meals, moving within the community, cleaning and maintaining the house,)<br>☐ Transportation<br>☐ Health care (doctor visits, medications)<br>☐ Supervision<br>☐ Emotional support<br>☐ Caring for their children or other dependents<br><br>☐ Other (please describe):<br>____________________________________<br>____________________________________<br>____________________________________
14. Select any of the following items that apply:	☐ I was recently hospitalized (or had a recent health crisis)<br>☐ Caregiving will likely continue indefinitely<br>☐ Care recipient is at risk for institutionalization<br>☐ Care recipient requires assistance with three or more personal care tasks (see question 13)<br>☐ My income is at or below federal poverty level<br>☐ None of the statements apply to me<br>☐ Prefer not to answer

15. Surgical information:	a. Procedures performed	☐ Arthroscopy<br>☐ Labral Repair<br>☐ Bone Trimming<br>☐ Loose Body Removal<br>☐ Other ________________<br>☐ Unknown
	b. Patient's fitness level before surgery (hrs of moderate activity/exercise)<br><br>Moderate intensity activity is where you notice your heart rate and breathing get faster than at rest, but you are still able to carry a conversation.	☐ Low (<1 hrs/week)<br>☐ Medium (1-3 hrs/week)<br>☐ High (>3 hrs/week)
	c. Time since procedure (days):	____________
	d. Were there complications after surgery:	☐ Yes (please describe):<br>______________________<br>______________________<br>______________________<br>☐ No
16. Patient ambulatory status	a. Weight-bearing status	☐ Non-weight bearing<br>☐ Partial weight bearing<br>☐ Full weight bearing

	b. Mobility aids used	☐ None (ambulatory)<br>☐ Wheelchair<br>☐ Walker<br>☐ Two crutches<br>☐ One crutch<br>☐ Cane<br>☐ Other (specify):<br><br>________________________
17. Does the care recipient have any other medical conditions? Select all that apply:	☐ Osteopenia<br>☐ Osteoporosis<br>☐ Osteoarthritis<br>☐ Back pain<br>☐ Previous lower extremity injury<br>☐ Rheumatoid arthritis<br>☐ Lung disease (COPD)<br>☐ Diabetes<br>☐ Stomach disease/Ulcers<br><br>☐ Kidney disease<br>☐ Anemia<br>☐ Depression<br>☐ Cancer<br>☐ Heart disease<br>☐ High Blood Pressure<br>☐ Genitourinary<br>☐ Dementia<br>☐ Other: (please list below)<br><br>____<br>____<br>____<br>____	

18. Please list three things that make caregiving challenging

1. ____

2. ____

3. ____

19. Please list three things that the healthcare team can do to make caregiving easier for you

1. ____

2. ____

3. ____
