# Supplementary material for: Moderate burden amongst caregivers posthip arthroscopy linked to younger caregiver age and task load: A cross‐sectional survey study
Source: Knee Surg Sports Traumatol Arthrosc. 2024 Aug 15;33(2):728–38. doi: 10.1002/ksa.12414 (PMC11792098; doi:10.1002/ksa.12414)
Supplement: Supplementary file 2 — Supporting information. [file KSA-33-728-s002.pdf]

Authors: The Caregiver Burden Survey Investigators
Corresponding Author: Dr. Olufemi R. Ayeni, McMaster University, ayenif@mcmaster.ca

Online Resource 2

Caregiver Burden Inventory Questionnaire

Choose the number that best represents how often the statement describes your feelings

0 – Never
1 – Rarely
2 – Sometimes
3 – Quite Frequently
4 – Nearly Always

Time Dependency Items	0	1	2	3	4
He/she needs my help to perform many daily tasks					
He/she is dependent on me					
I have to watch him/her constantly					
I have to help him/her with many basic functions					
I don't have a minute's break from his/her chores					

Development Items	0	1	2	3	4
I feel that I am missing out on life					
I wish I could escape from this situation					
My social life has suffered					
I feel emotionally drained due to caring for him/her					
I expected that things would be different at this point in my life					

Physical Health Items	0	1	2	3	4
I'm not getting enough sleep					
My health has suffered					
Care giving has made me physically sick					
I'm physically tired					

Emotional Health Items	0	1	2	3	4
I feel embarrassed over his/her behaviour					
I feel ashamed of him/her					
I resent him/her					
I feel uncomfortable when I have friends over					
I feel angry about my interactions with him/her					

Social Relationships Items	0	1	2	3	4
I don’t get along with other family members as well as I used to					
My care giving efforts aren’t appreciated by others in my family					
I’ve had problems with my marriage (or other significant relationship)					
I didn’t get along as well as I used to with others					
I feel resentful of other relatives who could but do not help					

**Total Score:**	
