# Supplementary material for: Moderate burden amongst caregivers posthip arthroscopy linked to younger caregiver age and task load: A cross‐sectional survey study
Source: Knee Surg Sports Traumatol Arthrosc. 2024 Aug 15;33(2):728–38. doi: 10.1002/ksa.12414 (PMC11792098; doi:10.1002/ksa.12414)
Supplement: Supplementary file 3 — Supporting information. [file KSA-33-728-s001.docx]

**Online Resource 3**

**Supplemental Digital Content – Supplemental Tables**

**Table S1**. Prognostic Factors for Caregiver Burden and their Hypothesized Effect

| **Variable** | **No. of levels** | **Hypothesized Effect** |
| --- | --- | --- |
| Caregiver age | 1 | Literature is mixed. ^1,4^ We hypothesize that in this population, higher age will be associated with lower caregiver burden (-). |
| Caregiver gender  Male  Female (+) | 1 | Women sex associated with poor self-perceived mental health in a meta-analysis^1,2^, but not in other orthopedic studies^1^. We hypothesize that female sex will be associated with higher CBI. (+) |
| Employment status at time of survey  Currently working full-time or studying (+)  Not currently working (part-time working, retired, unable to work or unemployed)  *Combined: 0,5 (Currently working full-time or studying) versus 1,2,3,4 (Not currently working)* | 1 | Being employed was associated with more depression symptoms^5^.  Employment – full-time (+) |
| Caregiver’s parental status  Yes, any children under 18 years-old (+)  No children under 18 years-old | 1 | We hypothesize that having a child under 18 (another care receiver) will be associated with higher burden. (no reference) |
| Caregiver lives with recipient  Yes  No | **1** | By extrapolation^4,5^(+)  Co-residence associated with lower psychological well-being^5^ (+). However, another study after total hip arthroplasty showed the opposite, with higher levels of stress in caregivers living apart from care receiver^2^.^†^ |
| Number of comorbidities *(continuous)* | **1** | We hypothesize that more complex patients’ health status (more comorbidities) will be associated with higher burden. (no reference) |
| Patient’s fitness level before surgery (hrs of moderate activity/exercise)  Low (<1 hourss/week) to medium (1-3 hrs/week)  High (>3 hours/week)  **Choices 0 and 1 combined.* | **1** | A study showed significant correlation between low physical functioning status and higher burden^3^ (+). By extrapolation, we hypothesize that low and medium fitness levels will be associated with higher burden. |
| Weight-bearing status at the time of survey  Non-weight bearing  Partial or full weight bearing | **1** | By extrapolation (showed significant correlation between low physical functioning status and higher burden)^3^ |
| Frequency of provided care to recipient  More than once a day (+)  Once a day or less (*choices 1,2,3,4 combined)* | **1** | Lower level of care provision was associated with greater well-being ^5^ |
| Caregiving tasks to recipient (number of) | **1** | Number of caregiving tasks was associated with more depression symptoms and less psychological well-being^5^. |
|  | **10** |  |

CBI=Caregiver Burden Inventory; ^†^scenario wherein evidence is mixed; (+) greater caregiver burden; (-) lower levels of caregiver burden

**Table S2.** Association Between the Time Dependency Score and Characteristics of the Caregiver, the Care Receiver and the Caregiving Episode

| **Predictor** | **Beta** | **95% CI** | **p-value** |
| --- | --- | --- | --- |
| **Caregiver age** | -0.095 | -0.183, -0.007 | **0.035** |
| **Caregiver gender** |  |  |  |
| Male | — | — |  |
| Female | -0.008 | -1.79, 1.78 | >0.99 |
| **Frequency of care provided** |  |  |  |
| More than once a day | — | — |  |
| Once a day or less | -1.75 | -4.55, 1.05 | 0.22 |
| **Caregiver has one or more children <18 years of age** |  |  |  |
| Yes | — | — |  |
| No | 0.206 | -1.60, 2.01 | 0.82 |
| **Caregiver’s employment status** |  |  |  |
| Currently working full-time or studying | — | — |  |
| Not currently working full-time | 1.24 | -0.753, 3.23 | 0.22 |
| **Patient’s fitness level before surgery** |  |  |  |
| Low (<1 hrs/week) to medium (1-3 hrs/week) | — | — |  |
| High (>3 hrs/week) | -0.309 | -2.16, 1.54 | 0.74 |
| **Weight-bearing status** |  |  |  |
| Non-weight bearing | — | — |  |
| Partial weight bearing | -2.08 | -4.11, -0.057 | **0.044** |
| **Caregiver lives with recipient** |  |  |  |
| Yes | — | — |  |
| No | 0.053 | -2.95, 3.06 | 0.97 |
| **Number of caregiving tasks provided** | 1.12 | 0.503, 1.73 | **<0.001** |
| **Number of comorbidities** | 0.307 | -0.171, 0.784 | 0.21 |
|  |  |  |  |
| ***Adjusted R^2^ = 0.218*** |  |  |  |
| CI=Confidence Interval; bolded=p-value<0.05 | | | |

**Table S3.** Association Between the Development Score and Characteristics of the Caregiver, the Care Receiver and the Caregiving Episode

| **Predictor** | **Beta** | **95% CI** | **p-value** |
| --- | --- | --- | --- |
| **Caregiver age** | -0.090 | -0.169, -0.011 | **0.027** |
| **Caregiver gender** |  |  |  |
| Male | — | — |  |
| Female | 1.80 | 0.194, 3.40 | **0.028** |
| **Frequency of care provided** |  |  |  |
| More than once a day | — | — |  |
| Once a day or less | -1.19 | -3.63, 1.26 | 0.34 |
| **Caregiver has one or more children <18 years of age** |  |  |  |
| Yes | — | — |  |
| No | -1.15 | -2.78, 0.471 | 0.16 |
| **Caregiver’s employment status** |  |  |  |
| Currently working full-time or studying | — | — |  |
| Not currently working full-time | 0.843 | -0.962, 2.65 | 0.36 |
| **Patient’s fitness level before surgery** |  |  |  |
| Low (<1 hrs/week) to medium (1-3 hrs/week) | — | — |  |
| High (>3 hrs/week) | 0.078 | -1.57, 1.73 | 0.93 |
| **Weight-bearing status** |  |  |  |
| Non-weight bearing | — | — |  |
| Partial weight bearing | -1.35 | -3.17, 0.463 | 0.14 |
| **Caregiver lives with recipient** |  |  |  |
| Yes | — | — |  |
| No | -0.055 | -2.78, 2.67 | 0.97 |
| **Number of caregiving tasks provided** | 0.449 | -0.102, 1.00 | 0.11 |
| **Number of comorbidities** | 0.206 | -0.223, 0.636 | 0.34 |
|  |  |  |  |
| ***Adjusted R^2^ = 0.133*** |  |  |  |
| CI=Confidence Interval; bolded=p-value<0.05 | | | |

**Table S4.** Association Between the Social Relationships Score and Characteristics of the Caregiver, the Care Receiver and the Caregiving Episode

| **Predictor** | **Beta** | **95% CI** | **p-value** |
| --- | --- | --- | --- |
| **Caregiver age** | -0.043 | -0.086, 0.000 | 0.052 |
| **Caregiver gender** |  |  |  |
| Male | — | — |  |
| Female | 0.604 | -0.267, 1.47 | 0.17 |
| **Frequency of care provided** |  |  |  |
| More than once a day | — | — |  |
| Once a day or less | -0.524 | -1.95, 0.900 | 0.47 |
| **Caregiver has one ore more children <18 years of age** |  |  |  |
| Yes | — | — |  |
| No | -0.188 | -1.09, 0.718 | 0.68 |
| **Caregiver’s employment status** |  |  |  |
| Currently working full-time or studying | — | — |  |
| Not currently working full-time | 1.34 | 0.309, 2.37 | **0.012** |
| **Patient’s fitness level before surgery** |  |  |  |
| Low (<1 hrs/week) to medium (1-3 hrs/week) | — | — |  |
| High (>3 hrs/week) | -0.235 | -1.13, 0.658 | 0.60 |
| **Weight-bearing status** |  |  |  |
| Non-weight bearing | — | — |  |
| Partial weight bearing | -0.601 | -1.59, 0.389 | 0.23 |
| **Caregiver lives with recipient** |  |  |  |
| Yes | — | — |  |
| No | -0.666 | -2.22, 0.889 | 0.40 |
| **Number of caregiving tasks provided** | 0.169 | -0.129, 0.467 | 0.26 |
| **Number of comorbidities** | 0.180 | -0.053, 0.413 | 0.13 |
|  |  |  |  |
| ***Adjusted R^2^ = 0.126*** |  |  |  |
| CI=Confidence Interval; bolded=p-value<0.05 | | | |

**Table S5.** Association Between the Physical Health Score and Characteristics of the Caregiver, the Care Receiver, and the Caregiving Episode

| **Predictor** | **Beta** | **95% CI**^1^ | **p-value** |
| --- | --- | --- | --- |
| **Caregiver age** | -0.053 | -0.138, 0.033 | 0.22 |
| **Caregiver gender** |  |  |  |
| Male | — | — |  |
| Female | 1.23 | -0.505, 2.97 | 0.16 |
| **Frequency of care provided** |  |  |  |
| More than once a day | — | — |  |
| Once a day or less | -1.87 | -4.56, 0.826 | 0.17 |
| **Caregiver has one or more children <18 years of age** |  |  |  |
| Yes | — | — |  |
| No | -0.791 | -2.54, 0.963 | 0.37 |
| **Caregiver’s employment status** |  |  |  |
| Currently working full-time or studying | — | — |  |
| Not currently working full-time | 0.378 | -1.57, 2.33 | 0.70 |
| **Patient’s fitness level before surgery** |  |  |  |
| Low (<1 hrs/week) to medium (1-3 hrs/week) | — | — |  |
| High (>3 hrs/week) | 0.067 | -1.73, 1.86 | 0.94 |
| **Weight-bearing status** |  |  |  |
| Non-weight bearing | — | — |  |
| Partial weight bearing | -0.876 | -2.84, 1.09 | 0.38 |
| **Caregiver lives with recipient** |  |  |  |
| Yes | — | — |  |
| No | 0.491 | -2.43, 3.41 | 0.74 |
| **Number of caregiving tasks provided** | 0.571 | -0.027, 1.17 | 0.061 |
| **Number of comorbidities** | 0.298 | -0.166, 0.762 | 0.21 |
|  |  |  |  |
| ***Adjusted R^2^ = 0.06*** |  |  |  |
| CI=Confidence Interval; bolded=p-value<0.05 | | | |

**Table S6.** Association Between the Emotional Health Score and Characteristics of the Caregiver, the Care Receiver and the Caregiving Episode

| **Predictor** | **Beta** | **95% CI** | **p-value** |
| --- | --- | --- | --- |
| **Caregiver age** | -0.001 | -0.012, 0.010 | 0.86 |
| **Caregiver gender** |  |  |  |
| Male | — | — |  |
| Female | 0.083 | -0.135, 0.301 | 0.45 |
| **Frequency of care provided** |  |  |  |
| More than once a day | — | — |  |
| Once a day or less | -0.127 | -0.478, 0.224 | 0.47 |
| **Caregiver has one or more children <18 years of age** |  |  |  |
| Yes | — | — |  |
| No | -0.159 | -0.380, 0.062 | 0.16 |
| **Caregiver’s employment status** |  |  |  |
| Currently working full-time or studying | — | — |  |
| Not currently working full-time | 0.093 | -0.153, 0.340 | 0.45 |
| **Patient’s fitness level before surgery** |  |  |  |
| Low (<1 hrs/week) to medium (1-3 hrs/week) | — | — |  |
| High (>3 hrs/week) | 0.005 | -0.218, 0.229 | 0.96 |
| **Weight-bearing status** |  |  |  |
| Non-weight bearing | — | — |  |
| Partial weight bearing | -0.028 | -0.276, 0.219 | 0.82 |
| **Caregiver lives with recipient** |  |  |  |
| Yes | — | — |  |
| No | -0.112 | -0.487, 0.262 | 0.55 |
| **Number of caregiving tasks provided** | -0.003 | -0.078, 0.072 | 0.94 |
| **Number of comorbidities** | 0.051 | -0.008, 0.109 | 0.089 |
|  |  |  |  |
| ***Adjusted R^2^ = -0.025*** |  |  |  |
| CI=Confidence Interval; bolded=p-value<0.05 | | | |

**Table S7.** Representative Quotes for Factors that Make Caregiving Challenging

| **Reported Factors** | **Representative Quotes** |
| --- | --- |
| Time management challenges | “Disruptive (hard to get things done)”  “Having to ask for days off from work“ |
| Taking care of other family members | “Having small children“  “Other family responsibilities” |
| Physical and emotional strain of caregiving | “The physicality of it, lifting her up and down“  “Getting her up to put on compression socks“ |
| Additional responsibilities and task overload | “Household chores“  “Prepping meals” |
| Emotional support, coaching, and patient's mood management | “Entertaining/distracting her“  “Emotional support, patient is confined initially and requires attention” |
| Home environment challenges | “Sleeping on a different level“  ““Arranging the house to accommodate her needs” |
| Personal health and limitations of caregivers | “Personal health and limitations of caregivers”  “My own fatigue (I have cancer)” |
| Managing medications and medical equipment | “Stress of ‘meds’ (correct ‘admin’)“  “Finding equipment (i.e., portable ice machine)” |
| Lack of information or education on management | “Knowing if we were positioning her properly”  “Not knowing what is normal and what is not“ |
| Challenges with personal hygiene and intimacy | “Bathing, dressing up, going to the washroom“  “Gender difference with child“ |
| Sleep disruption | “Lack of sleep“  “Being needed all hours of night to assist“ |
| Financial and logistical challenges | “Cost - expensive to rent machines, pay for transportation (cause unable to get him in/out of our cars)”  “Financially” |
| Challenges with hospital discharge and travel home | “Bringing patient home in pain/on drugs/on crutches”  “Getting her home was traumatic“ |
| Isolation and lack of support | “Other people understanding her limitations”  “Lack of understanding (family/friends)“ |

**Table S8.** Representative Quotes for Factors that the Healthcare Team Can Do to Facilitate Caregiving

| **Reported Factors** | **Representative Quotes** |
| --- | --- |
| More detailed communication and information transfer in general | “Explain everything in writing for reference purposes“  “Follow-up call with nurse/doc for questions“ |
| Extended hospital stays | “Allow for optional overnight stay for 1st night”  “Keep hospitalized until heavier medications are finished” |
| Facilitated access to home care assistance and services | “Provided housekeeping help“  “Faster referral to home healthcare” |
| More education on physical rehabilitation, restrictions, and medical equipment | “Appointment prior to surgery with someone who can show the caregiver and patient how to move during the first week when not weight bearing”  “More info on exercises to do with feet for blood flow“ |
| Facilitated access to medical equipment | “Assistance with obtaining aids: crutches, toilet seat, etc.“  “Provide the crutches, devices needed at hospital“ |
| More education on medication | “Recommending a clear schedule on consuming pain meds”  “Provide prescription prior to surgery for meds to allow less running for caregiver with patient” |
| Financial and economic assistance | “Give assistance for payment of hospital bed ‘and’ other equipment needed”  “’Rehabilitation’ coverage” |
| Optimize discharge | “Better proof wraps for bandages“  “Leaving the hospital was an issue - safely getting into the vehicle” |
| Better efficiency of care and follow-up | “Flexible appointment times“  “Minimize long waiting times“ |
| Encouragement for patient cooperation | “Make the patient listen“ |
| Other/Unrelated | “Not sure”  “I feel as though the health team has done everything they can. They make this experience as easy as possible already” |

**REFERENCES**

1. Ariza-Vega P, Ortiz-Piña M, Kristensen MT, Castellote-Caballero Y, Jiménez-Moleón JJ. High perceived caregiver burden for relatives of patients following hip fracture surgery. *Disabil Rehabil*. 2019;41(3):311-318.

2. Chow WH. An Investigation of Carers’ Burden: Before and after a Total Hip Replacement. *Br J Occup Ther*. 2001;64(10):503-508.

3. Lin PC, Lu CM. Psychosocial Factors Affecting Hip Fracture Elder’s Burden of Care in Taiwan. *Orthop Nurs*. 2007;26(3):155-161.

4. Pinquart M, Sörensen S. Differences between caregivers and noncaregivers in psychological health and physical health: A meta-analysis. *Psychol Aging*. 2003;18(2):250-267.

5. Pinquart M, Sörensen S. Spouses, adult children, and children-in-law as caregivers of older adults: A meta-analytic comparison. *Psychol Aging*. 2011;26(1):1-14.
